# Comparative genomics reveals convergent rates of evolution in ant–plant mutualisms

**DOI:** 10.1038/ncomms12679

**Published:** 2016-08-25

**Authors:** Benjamin E. R. Rubin, Corrie S. Moreau

**Affiliations:** 1 Committee on Evolutionary Biology, University of Chicago, 1025 East 57th Street, Culver Hall 402, Chicago, Illinois 60637, USA; 2 Department of Science and Education, Integrative Research Center, Field Museum of Natural History, 1400 South Lake Shore Drive, Chicago, Illinois 60605, USA

## Abstract

Symbiosis—the close and often long-term interaction of species—is predicted to drive genome evolution in a variety of ways. For example, parasitic interactions have been shown to increase rates of molecular evolution, a trend generally attributed to the Red Queen Hypothesis. However, it is much less clear how mutualisms impact the genome, as both increased and reduced rates of change have been predicted. Here we sequence the genomes of seven species of ants, three that have convergently evolved obligate plant–ant mutualism and four closely related species of non-mutualists. Comparing these sequences, we investigate how genome evolution is shaped by mutualistic behaviour. We find that rates of molecular evolution are higher in the mutualists genome wide, a characteristic apparently not the result of demography. Our results suggest that the intimate relationships of obligate mutualists may lead to selective pressures similar to those seen in parasites, thereby increasing rates of evolution.

## Abstract (web summary)

Mutualisms in which ants protect plants in exchange for food and shelter have arisen independently multiple times. Here, Rubin and Moreau sequence the genomes of three mutualistic ant species and four of their non-mutualistic relatives and show that the transition to mutualism is associated with elevated evolutionary rates across the genome

## Graphical abstract

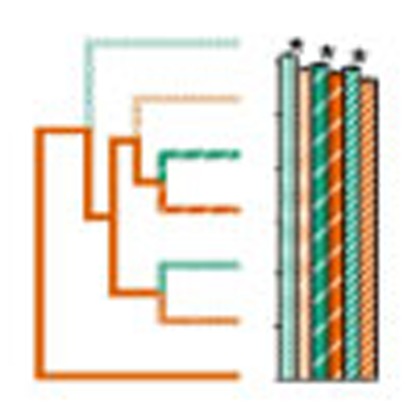

Mutualisms are ubiquitous in nature and profoundly influence the structure of ecosystems^1^,^2^. In addition to influencing the ecology of the organisms involved, these intimate interactions can have radical consequences on genome evolution^3^,^4^. Van Valen^5^ predicted increased rates of molecular evolution in antagonistically coevolving species with his Red Queen Hypothesis, a theory that has gained much support, particularly in studies of loci involved in disease and resistance^6^,^7^. Far less research has examined the influence of mutualisms on rates of evolution; however, some theory has suggested an advantage for mutualists to evolve more slowly than their partners^8^,^9^,^10^. This Red King effect^8^ results from selection for greater concessions from at least one of the two members of a mutualism. Although both are initially selected to forfeit additional resources to their partner, the more slowly evolving species maintains selfish behaviour for longer, gaining an advantage. This outcome is highly dependent on the starting conditions of the relationship and in a more limited subset of conditions higher rates of change are instead predicted to be advantageous. However, verbal models also predict selection to favour the more slowly evolving species: each member of the mutualism is adapted to draw maximum benefit from the most common of its partners' phenotypes, thereby selecting against changes^9^.

Ant–plant mutualisms, wherein ants nest obligately in chambers provided by their host plants while protecting them from herbivores, have evolved in a number of ant–plant pairs^11^,^12^,^13^,^14^. The ant genus *Pseudomyrmex* includes mutualistic acacia–ants^11^,^14^ as well as two other clades that nest not in the hollow thorns of acacias but in the trunks and leaf petioles of distantly related plants in the genera *Triplaris* and *Tachigali*, respectively. These three clades of ants have evolved mutualism convergently, despite a remarkable similarity in behaviour^12^,^13^; all *Pseudomyrmex* plant–ant mutualists are incredibly aggressive, actively patrolling and attacking herbivores and invaders^11^,^12^,^13^,^14^. Non-mutualistic species of *Pseudomyrmex*, the generalists, live in the same environments, yet have starkly different behaviour, fleeing from aggressors even when their own nests are at risk^11^,^12^,^15^.

Leveraging these three evolutionarily independent clades of closely related mutualists (the entire clade diverged <10 Mya^16^ and shows <5% sequence divergence^13^,^16^), we sought to understand the evolution of this behavioural difference through full genome sequencing. We first *de novo* sequenced the genome of a single species of *Pseudomyrmex*. We then used this reference to assemble and align the genomes of six other species: three mutualists and three generalists. These full genome sequences reveal that mutualists have higher rates of molecular evolution than generalists genome wide, revealing unexpected consequences of beneficial symbioses. In addition, we identify a number of genes showing convergent signatures of positive selection in mutualists, many of which are involved in neurological processes, a probable target of selection for mutualistic behaviours.

## Results

### *Pseudomyrmex gracilis* genome assembly

The most closely related species for which genomic resources exist are separated from *Pseudomyrmex* by ∼100 million years^16^. Therefore, we developed a novel reference by *de novo* sequencing the genome of a single haploid male of the widespread generalist *P. gracilis.* Paired-end 100 base Illumina HiSeq sequencing yielded 358 million reads with overlap between paired reads of 52±15 (mean±s.d.). We obtained 318 million reads from a mate-pair library separated by 2,453±344 bases.

We assembled the *P. gracilis* genome with ALLPATHS-LG (ref. 17) and our final assembly consists of 24,064 contigs on 6,556 scaffolds with a total coverage of 89X. Total contig and scaffold lengths are 261 and 282 Mb, respectively. Contig N50 is 30 kb and scaffold N50 is 350 kb. ALLPATHS-LG k-mer spectrum analysis estimated the genome size to be 392 Mb and, at a k-mer size of 25, genome repetitiveness to be 47%.

### Repetitive elements are abundant

A total of 2,519 repetitive elements were initially identified in the *P. gracilis* genome assembly (563 from PILER-DF (ref. 18), 1,956 RepeatModeler (http://www.repeatmasker.org)). After redundant sequences were removed, 1,607 repetitive elements remained. Of these, 42 sequences were below the length cutoff (<80 bases) and 77 more were removed due to similarity with known proteins. The final set of transposable elements (TEs) was composed of 1,488 sequences. Overall, 6.0% (16 Mb) of the assembled contigs are composed of simple repeats and 20.5% (53 Mb) are TEs (Supplementary Data 1). Given that overall genome repetitiveness was estimated to be nearly 50% and genome size was estimated to be more than 100 Mb longer than the assembly, most of the unassembled sequence is likely composed of repetitive elements.

### Pervasive introgression of mitochondrial genome

Introgressions of mitochondrial genes into the nuclear genome are common in *P. gracilis.* We identified at least one copy of 11 mitochondrial genes and a total of 228 mitochondrial-like genes in the *P. gracilis* assembly compared with 173 in the ant *Atta cephalotes* and 97 or fewer in six other ant genomes (Supplementary Table 1).

### Linkage mapping places scaffolds on chromosomes

We created a linkage map by genotyping 47 workers and the queen from a single colony using reduced representation Genotyping-By-Sequencing (GBS)^19^. From this library, between 836,984 and 6,115,576 reads were assigned to each sample (Supplementary Data 2) for a total of 167 million usable reads. Of these, 134 million (80%) reads passed quality filtering. Clustering at 90% similarity yielded between 120,262 and 231,138 clusters per sample. Mean within sample cluster depth ranged from 5.0 to 13.3 (24.4±3.1). The mean error and heterozygosity rates as calculated by pyRAD^20^ were 0.15% and 0.88%, respectively. We recovered a total of 31,371 loci with data for at least 24 samples after between sample clustering. Each sample had data for between 3,952 and 30,442 (26,362±4,448) loci. The individual with the smallest number of loci was a distinct outlier and was excluded from further analyses. At least one site in which a single-nucleotide polymorphism was represented by at least two samples was present in 5,216 loci. Our stringent quality filtering reduced this number to 565 loci used in linkage mapping analysis.

After determining the correct phase^21^, MSTmap^22^ produced 38 syntenic linkage groups with at least three loci. Previous karyotype analyses have shown the presence of 35 chromosomes in *P. gracilis*^23^. Our linkage groups therefore probably correspond broadly to chromosomes. No scaffold appeared in more than one linkage group; thus, we were able to confidently assign 185 scaffolds to our 38 linkage groups (Supplementary Data 3).

### Coding sequence annotation

We sequenced messenger RNA from a variety of life stages of *P. gracilis* to assist with gene annotation. From a 100 base paired-end Illumina RNA sequencing library, we obtained 204 million reads and 174 million of these passed quality filtering. We assembled the transcriptome from these data resulting in 204,150 transcripts at least 200 bp long. Similarity clustering reduced the number of sequences to 176,795.

Our final annotation using the automated pipeline MAKER^24^ consisted of 16,069 genes, including 367 *in silico* predictions that were identified by InterProScan^25^ as probable genes. This is within the range that we see in social Hymenoptera and within 100 genes of other ants^26^.

Of the 248 ultra-conserved CEGMA^27^ genes, 247 were at least partially present in the assembly and 244 (98%) of these were complete. We found evidence for 77 of the 79 (98%) minimum expected cytoplasmic ribosomal proteins in our final gene set, suggesting that the annotation was relatively complete. The two missing CRPs are both quite short (RPL29: 76 AAs in *Drosophila* and RPL39: 51 AAs) and may have been missed partly for this reason. We did find signs of these two genes in the genome assembly, indicating a limitation of the annotation rather than the assembly.

### Enrichment for ATP synthesis genes in *P. gracilis*

There are 5,697 *P. gracilis* genes without clear orthologues in any other species. Relative to the 10,372 genes with orthologues, these genes are enriched in a number of ontology terms related to ATP synthesis (Supplementary Data 4). Given the clear presence of NuMts in the *P. gracilis* genome, we were concerned that the apparent increased number of cellular respiration related genes was the result of spurious annotation of pseudogenes with mitochondrial origins. We confirmed that these genes were, at least, transcriptionally active by comparing the set of genes classified in enriched categories with the independently assembled transcriptome using BLASTN. Of the 713 genes classified to enriched categories, 367 (52%) are 99% identical to assembled transcripts across at least 25% of the length of both the gene and the transcript. By comparison, 11,349 of the 16,069 (71%) total genes show the same pattern. Although the genes unique to *P. gracilis* that do not correspond directly to assembled transcripts could still be functional genes, this difference in well-supported genes was concerning. We therefore re-ran the analysis with only those genes directly supported by transcripts. Of the genes unique to *P. gracilis*, 2,953 (52%) were directly supported by transcripts and no Gene Ontology (GO) terms were found to be enriched in this reduced set of genes. It is, therefore, unclear whether the genes classified in the enriched GO categories are functional. However, they could certainly be transcribed at low levels and not picked up by our RNA sequencing. The enriched GO categories found by comparing *P. gracilis* to the other Hymenopteran species are similar to those enriched within *P. gracilis* (Supplementary Data 5).

### Majority of ingroup genomes recovered by reference mapping

**Figure 1:**
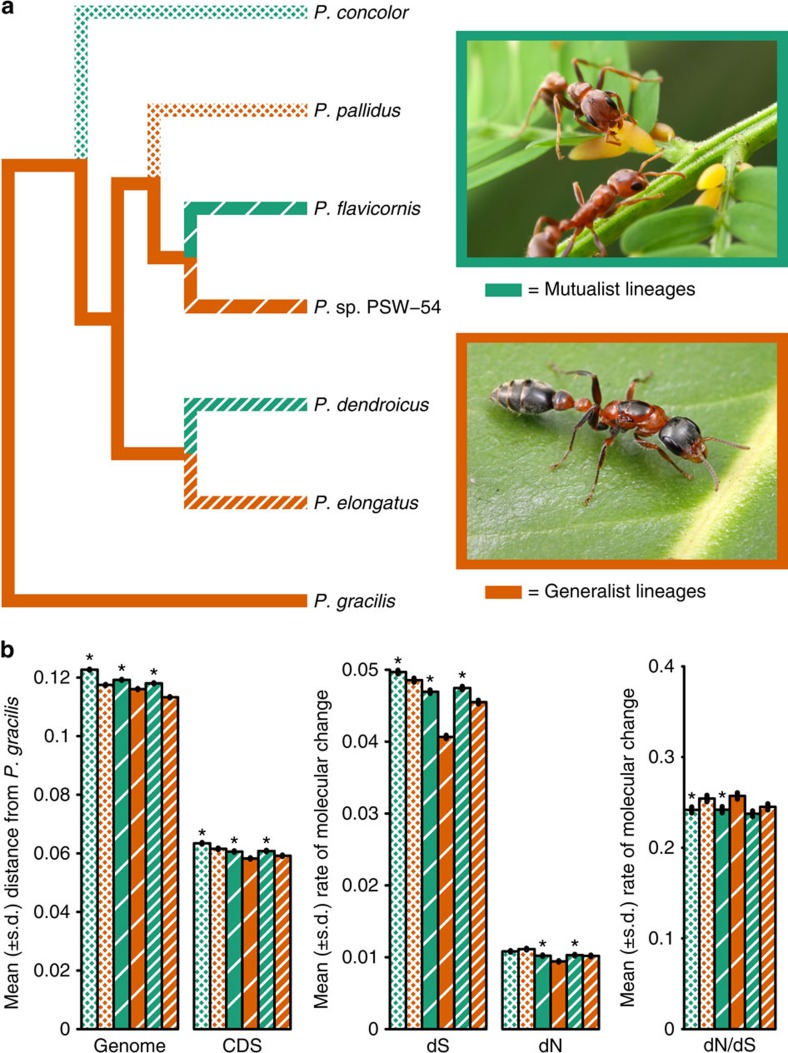
Convergent increase in rates of genome evolution in mutualists. (**a**) Phylogenetic relationships between species sequenced in this study. Throughout, green indicates mutualists and orange indicates generalists. Acacia–ant mutualist *Pseudomyrmex spinicola* and generalist *P. gracilis* are pictured. Photographs ©Alex Wild/alexanderwild.com and used with permission. (**b**) Mean±s.d. of genetic distances of each species from *P. gracilis* in 38,862 25 kb windows across the genome and limited to 4,082 coding regions (CDS). Estimates of dN, dS and dN/dS are also shown. Significance (*P*<0.01) was determined between species with paired *t*-tests across all windows and Wilcoxon signed-rank tests across all genes. We indicate significance only between the pairs of species: *P. concolor* versus *P. pallidus*, *P. flavicornis* versus *P.* sp. PSW-54 and *P. dendroicus* versus *P. elongatus*. All other significance values are shown in Supplementary Table 3.

We used our *P. gracilis* genome as a reference to assemble the genomes of a representative species from each of the three clades of *Pseudomyrmex* mutualists (*P. concolor*, *P. dendroicus* and *P. flavicornis*) and three of the most closely related generalists (*P. pallidus*, *P. elongatus* and the undescribed species *P.* sp. PSW-54) (Fig. 1a). Using this approach, we were able to assemble aligned sequence for approximately two-thirds of the total genome length and 11,052 genes from all 7 species (Supplementary Table 2).

### Full genomes resolve *Pseudomyrmex* phylogeny

The topology that we inferred was slightly different than what we expected based on previous work^13^. In our tree, *P. pallidus* is most closely related to the clade containing *P. flavicornis* and *P.* sp. PSW-54 rather than *P. concolor* (Fig. 1). All nodes in this inference had bootstrap support of 100%.

We also reconstructed a total of 38,862 phylogenies from 25 kb sliding windows across the length of the genomes, yielding 29 distinct topologies (Supplementary Data 6). The most common topology in this set was identical to the concatenated whole genome topology and occurred 22,919 (59%) times. The topology from Ward and Downie^13^ occurred just a single time. Three other topologies were common, occurring 7,114, 4,145 and 3,426 times. All topologies that occurred at least ten times placed *P. dendroicus* sister to *P. elongatus* and *P. flavicornis* sister to *P.* sp. PSW-54, but the other two ingroup species moved around the base of these clades.

### No signatures of parallel evolution in mutualists

We searched for signs of parallel evolution in the mutualists using Saguaro^28^. None of the 40 resulting phylogenetic topologies showed the three mutualistic lineages as the most closely related. These results indicate that parallel evolution of particular alleles related to mutualistic behaviour from an ancestral population is unlikely to be responsible for the convergence of behaviour. Similarly, none of the phylogenies reconstructed from sliding windows had monophyletic clades of all three mutualists and no generalists.

### Rates of molecular evolution are higher in mutualists

**Figure 2:**
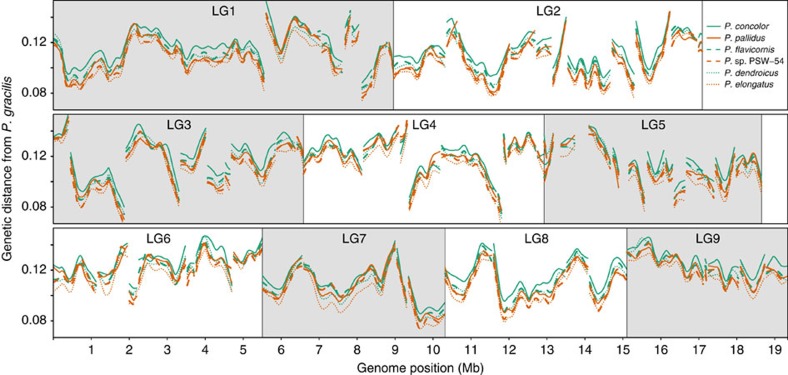
Elevated rates of molecular evolution are genome-wide. Genetic distance (proportion of segregating sites) of each ingroup species from *P. gracilis* in 100 kb sliding windows across the largest nine linkage groups (LG). Curves were smoothed with the R function lowess. The breaks in lines within linkage groups show the ends of scaffolds. It is noteworthy that the mutualists (green) tend to have greater genetic distance from *P. gracilis* when compared with the most closely related generalists (orange). Line textures show specific species.

We find that all three mutualists have significantly higher rates of molecular evolution than the most closely related generalists. This pattern is persistent in both coding and non-coding regions across the genome (Figs 1b and 2, and Supplementary Tables 3 and 4), suggesting a fundamental difference in substitution rates between mutualists and generalists, genome wide.

We deliberately sampled taxa so that the most closely related pairs of mutualists and generalists could be directly compared, thus reducing the potentially confounding effects of evolutionary history. Despite the clear trends between pairs of related mutualists and generalists when using this framework, there is substantial variation in rates of change within each group and each individual mutualist does not have higher rates of change than every generalist. For example, although the mutualist *P. dendroicus* has greater average dS than the most closely related generalist *P. elongatus*, it has lower average dS than the generalist *P. pallidus*. This pattern exhibits the power of our approach; comparing closely related taxa reduces the confounding effects of independent evolutionary history. Given the variation in rates within behavioural groups, our findings could not be identified without examining behaviourally convergent taxa.

### Differences in evolutionary rate are not due to demography

Differences in rates of molecular evolution have been reported in a few other groups^29^,^30^,^31^,^32^, but these findings are usually attributable to the differential action of genetic drift resulting from short generation times and small effective population sizes. However, in plant–ants, molecular evolution will, if anything, be slowed by generation time, because these species establish large colonies over several years before producing reproductive individuals. In contrast, generalists tend to live in small, transient nests and begin reproducing soon after colony founding, several-fold faster than mutualists^14^,^15^.

Population size differences are less obvious, but we used G-PhoCS^33^ to estimate population sizes using whole genome data. We obtained a total of 8,482 neutral loci with sequences from at least four of six ingroup species. Each species was present in at least 6,362 of these loci. Based on the resulting estimates of *θ* (Supplementary Table 5), it does not appear that there are consistent differences in population sizes between mutualists and generalists. Although *θ* is larger in *P.* sp. PSW-54 than in *P. flavicornis*, the other two pairs of mutualists and generalists show either the opposite pattern or are equivocal. The lower overall dN/dS ratios in mutualists also suggest larger population sizes (Fig. 1b and Supplementary Table 3). Population size is, therefore, unlikely to be responsible for the differences in rates of molecular evolution in these species.

### Relaxed selection may contribute to higher rates

The higher rates of molecular evolution in mutualists are, therefore, most likely to be due to a combination of relaxed purifying and positive selection rather than demography. Relaxed selection is responsible for rapid changes in the genomes of other obligate symbionts^3^,^4^, although, unlike these intracellular bacteria, plant–ants must maintain most of their genomic integrity. While the lower average dN/dS ratios in mutualists suggest that relaxed selection is not solely responsible for their elevated rates of evolution (Fig. 1b and Supplementary Tables 3 and 4), this observation must be tempered by the potential confounding effects of higher dS in mutualists. We, therefore, used repetitiveness as an additional proxy for relaxed selection.

If the higher rates of change in mutualistic genomes are due simply to large-scale relaxed selection across the genome, repetitiveness and TEs would also be expected to spread, as their small cost would be selected against less efficiently^34^. Therefore, we also evaluated the degree of relaxed selection by examining genomic repetitiveness. K-mer spectrum analysis shows that mutualists tend to have lower overall repetitiveness than generalists, although there is no consistent trend (Supplementary Table 2). However, the diversity of TEs is consistently lower in mutualists with a higher maximum abundance of individual TE families. The frequently dramatic deleterious consequences of ectopic recombination predict stronger selection against TE insertions from more abundant TE families^35^. The TE landscape in *Pseudomyrmex* mutualists is, therefore, suggestive of a slight relaxation of selection.

### DNA-repair protein changes may affect mutation rate

We attempted to identify genes that might be directly responsible for the elevated rates of molecular evolution in mutualists by searching for signs of relaxed selection and convergence in genes associated with DNA replication and repair. Fifty-two *P. gracilis* genes were classified as involved in DNA repair (GO:0006281) and 54 were classified as part of DNA replication (GO:0006260) by InterProScan. One DNA repair protein (PG04199: *gluon*) and two DNA replication proteins (PG00508: *mutagen-sensitive 205* and PG08332: *mitochondrial DNA helicase*) have consistently higher dN/dS ratios in mutualists, potentially indicating high rates of change in these genes. At site 433 of *gluon*, the mutualists *P. dendroicus* and *P. flavicornis* have converged on the negatively charged aspartic acid, whereas all other species have the polar but uncharged asparagine. At site 1,068, the mutualists *P. concolor* and *P. flavicornis* have the amino acid threonine, whereas all other species have alanine. Threonine has a polar side chain, whereas alanine is hydrophobic. However, according to Phyre2 (ref. 36) models, these convergent sites are not within active or binding regions of the gene. Similar convergent sites between generalists are not present in this gene.

Two sites within *mutagen-sensitive 205* are convergent between *P. dendroicus* and *P. concolor* (1,131 and 1,142, respectively). These species have the hydrophobic leucine and the positively charged lysine at these sites, respectively, whereas all other species have structurally rigid prolines and positively charged arginines. Neither of these sites is within or near the predicted binding or active sites. One site is similarly convergent within generalists.

Lastly, two sites within *mitochondrial DNA helicase* show patterns of mutualistic convergence. At site 23, both *P. dendroicus* and *P. flavicornis* have cysteine, whereas all other species have the positively charged arginine. At site 631, *P. dendroicus* and *P. concolor* possess an apparent insertion of leucine. However, all seven species have identical amino acids in and around the ATP-binding site and Walker motifs predicted by Phyre2.

Relaxing the quality controls slightly to include ambiguous data suggests that the DNA repair gene *Rev1* (PG04546) also has consistently higher dN/dS ratios in mutualists. Two sites are also convergent in mutualists within this gene. At site 149, *P. concolor* and *P. dendroicus* have valine as opposed to alanine, although these are structurally quite similar. At site 628, *P. concolor* and *P. flavicornis* have the polar threonine rather than the hydrophobic alanine. One site is similarly convergent in two generalists. In addition, each mutualistic lineage has an amino acid that is different from all other species within or immediately surrounding the predicted active site of *Rev1*, where it binds with *DNA polymerase eta*.

### Gene duplications more frequently convergent in generalists

Higher molecular evolutionary rates could influence rates of gene duplication. There were 19 transcribed regions with apparently higher copy numbers in mutualists and 44 in generalists (Supplementary Data 7). Nine and 22 of the genes with higher copies in mutualists and generalists, respectively, had readily identifiable orthologues in *Drosophila*.

### Small number of genes strongly selected in mutualists

We used both the branch test and branch-site test implemented in PAML version 4.7 (ref. 37), to test for signatures of positive selection within the mutualistic lineages. Of the 11,052 genes that passed completeness quality controls, 6,556 were at least 300 codons long. Among these, we found ten genes with branch-site signatures of positive selection within mutualists. Four of these also showed signatures of positive selection when tested on the generalist lineages, suggesting that this significance was spurious, probably due to high rates of evolution in these genes. Of the six remaining genes, three have experimentally determined functions. *Subito* is involved in structural arrangement of chromosomes during DNA replication, *tenectin* is involved in various aspects of morphogenesis and *myosin heavy chain* is responsible for muscle function (Supplementary Data 8).

The branch test for selection was significant for positive selection in mutualists in 17 genes (Supplementary Data 8). Eight of these have at least one experimentally determined function and three of these have roles in the nervous system, including neurogenesis and neurotransmission. Given their influence on the structure and function of the nervous system, changes in these genes are likely to be related to the extreme behavioural differences between mutualists and generalists.

Of the 4,082 genes for which we had high confidence estimates of dN and dS, 376 had consistently higher dN/dS ratios in mutualists and 772 had higher ratios in generalists. No GO terms were significantly enriched in either the genes with consistently higher dN/dS ratios in mutualists or generalists (*P*>0.05).

### Several sites in myosin heavy chain congergent in mutualists

Five amino acid sites within *myosin heavy chain* are convergent in at least two mutualists. Two of these sites are convergent across all mutualists (779 and 1,417). Site 1,417 is only a difference between two amino acids with similarly polar side chains (serine and asparagine), but the difference at site 779 is between a polar tyrosine in generalists and a hydrophobic phenylalanine in all three mutualists. Although neither substitution falls at a site specifically predicted to be functionally active, site 779 is well within the putative motor domain of the protein.

### Protein convergence in both mutualists and generalists

Of 5,906,079 amino acid sites examined, 4,283 (0.073%) were convergent between at least two mutualists and 4,110 (0.070%) between at least two generalists. These convergent sites were distributed well across 2,871 and 2,710 genes in mutualists and generalists, respectively. There were 196 sites (0.0033%) convergent between all three mutualists and 224 (0.0038%) between all three generalists. These sites were distributed across 178 and 206 genes in mutualists and generalists, respectively. Within mutualists, one gene (PG08867) has three completely convergent sites and another (PG15667) has four. Neither of these genes have clear *Drosophila* orthologues, although PG08867 was classified as a metallo-endopeptidase and PG15667 as a fatty acid desaturase by InterProScan. Two genes (PG01437 and PG11553) have three completely convergent sites within the generalists and one (PG03192) has six such sites. PG11553 and PG03192 also do not have clear orthologues but were classified as a ribonuclease and glycerophosphoryl diester phosphodiesterase by InterProScan. This leaves 13 genes with two completely convergent sites in mutualists and nine genes with two completely convergent sites in generalists (Supplementary Table 6). In mutualists, one of these genes is *myosin heavy chain*, as discussed above. *Stretchin-Mlck*, a gene involved in *myosin light chain* activity, is also in this gene set, providing stronger evidence that muscle function has changed in mutualists.

### Expression of fast-evolving genes is higher in heads

**Figure 3:**
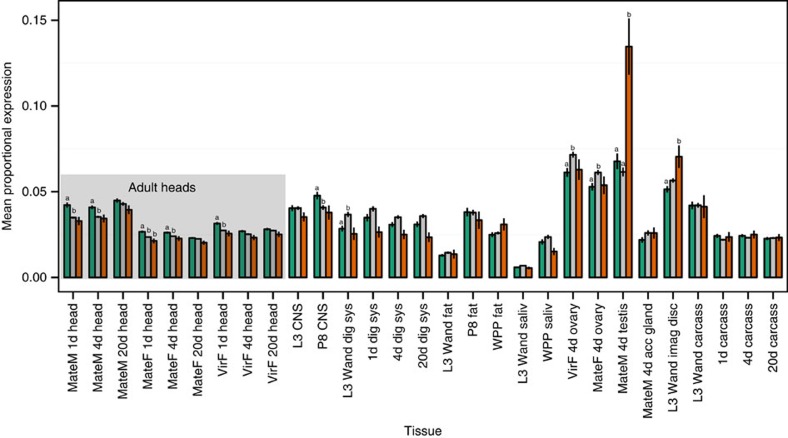
Genes evolving faster in mutualists are associated with the nervous system. Mean±s.e. expression levels of FlyBase orthologues of 4,727 genes with consistently greater dS in mutualists (green), generalists (orange) and all other genes (grey) for every tissue examined by modENCODE. False discovery rate-corrected significance is shown with letters. The large grey box highlights expression data from heads of adult flies.

Expression data from modENCODE provided further evidence of rapid evolution in genes related to the nervous system. We identified *Pseudomyrmex* orthologues in *Drosophila* for 6,477 genes and 4,727 of these were at least 300 bases long. Based on the modENCODE expression data, those genes with higher dS in mutualists tend to be expressed at higher levels in heads and lower levels in ovaries (Fig. 3 and Supplementary Data 9). The same trends apply to genes with higher dN (Supplementary Data 10), although the opposite appears true for genes with higher dN/dS (Supplementary Data 11). Pure genetic distance shows the clearest signal of higher expression in heads (Supplementary Data 12). This pattern may indicate weak selection on non-coding regions in these nervous system associated genes.

### Codon bias does not differ in mutualists

Although the mutualist *P. concolor* has significantly greater codon usage bias than all other taxa, the magnitude of genome-wide codon usage bias did not differ consistently between mutualists and generalists (Supplementary Table 3). All genomes show weak positive correlations between effective codon number and rate of non-synonymous substitution and negative correlations between effective codon number and rate of synonymous substitution estimated using the mutation-site models implemented in PAML^37^ (Supplementary Table 7). The same trends exist when using the physical-site models of HyPhy^38^, but the correlations are weaker and only rarely reach statistical significance, indicating that any true signal is minimal. Nevertheless, it appears that codon usage bias is positively correlated with synonymous substitution rate, possibly suggesting that selective forces for increased codon bias are active in these species. The negative correlation between non-synonymous substitution and codon usage probably indicates that genes experiencing relaxed selection in coding sequence are also released from selection on codon usage and are expressed at lower levels.

Although codon bias does not differ between mutualists and generalists, codon usage differs strongly, apparently as a result of the reduced GC content in mutualists (Supplementary Table 2). Third codon position GC content is lower in all mutualists than all generalists (Supplementary Table 2) and there is a concordant difference in codon usage (Supplementary Data 13). Synonymous codons with lower GC content are used in greater frequency in mutualists in almost every case (Supplementary Data 13). This difference in codon usage may simply be a result of mutational bias and the higher rate of change in mutualists.

### dN/dS ratios are not confounded by outliers

Although dN/dS ratios calculated from the sums of all dN and dS estimates for each species were lower than the means of dN/dS ratios, the differences in ratios between species remained consistent. Therefore, the differences in rates of molecular evolution are probably not an artefact due to outlying loci in particular taxa. The estimates of dN/dS based on sums are as follows: 0.218 for *P. concolor*, 0.229 for *P. pallidus*, 0.218 for *P. flavicornis*, 0.232 for *P.* sp. PSW-54, 0.217 for *P. dendroicus* and 0.224 for *P. elongatus*.

### No signatures of positive selection on aggression genes

We were able to find orthologues and calculate accurate dN/dS ratios for 37/51 genes from the short list of genes involved in aggression and 911/1,922 genes from the long list. Of the 383 genes with consistently higher dN/dS ratios in mutualists, 343 have clear honey bee orthologues and only one is from the short list and 60 from the long list. Of the 779 genes with higher dN/dS ratios in generalists, 709 have honey bee orthologues and two and 112 were identified in the short and long lists, respectively. This pattern suggests that, if anything, fewer of the genes related to aggression in bees are evolving faster in mutualists than is expected. The aggressiveness inherent in these mutualisms appears to be the result of changes in alternative mechanisms such as the expression levels examined in the original study^39^.

## Discussion

Elevated rates of molecular evolution appear to be typical in obligately symbiotic organisms including parasitic plants^30^ and lice^31^, suggesting the existence of a general evolutionary force on this type of life history strategy. The mechanisms causing these differences are often enigmatic but demography and other non-selective forces are typically invoked^30^,^31^,^32^. Rate changes resulting from selective forces are known from parasitic interactions^6^ but have yet to be reported in mutualists. Most genomic studies of mutualisms have focused on intracellular bacterial endosymbionts and, though these organisms have some of the highest known rates of genomic evolution^3^,^4^, the precipitous drop in population size and relaxed selection inherent in intracellular living make the separation of molecular mechanisms leading to rate changes difficult. The only other study to explore rates of molecular evolution among eukaryotic mutualists (lichenized fungi) also found evidence for increased rates among symbionts, though only 1,550 bp of ribosomal DNA were examined^32^. In this case the hypothesized explanation for the observed differences in rates is entirely non-biological: the increased exposure to solar radiation may increase mutation rate.

Theoretical models of interacting mutualistic species clearly show the existence of Red King effects^8^. Yet these models are simplified, focusing on the resources exchanged between two focal partners and do not include any influences from species external to the mutualism. We do not doubt the validity of these results in such an isolated system, but our study suggests influential additional factors. Firstly, though the degree of relaxation experienced by intracellular symbionts^3^,^4^ is unlikely to occur in less intimate interactions such as those explored here, it is likely be a common symptom of mutualism. Both the elevated rates of synonymous change and higher abundance of individual TE families in mutualist ants suggest that they experience some degree of genome-wide relaxed selection. Secondly, widespread positive selection is likely also involved, as suggested by the rates of change in nervous system-associated genes. Once locked into a mutualism, species must adapt not only to their own changing environments, but to those of their symbionts as well. Here, selection on the plants from outside the mutualistic system likely requires reciprocal adaptations from resident ant species and vice versa, a process much like what is seen in Red Queen interactions. For example, acacia trees produce sucrose-free nectar, making them less attractive to the majority of *Pseudomyrmex* species and, thereby, deterring exploitation by non-protective ants^40^. Mutualistic *Pseudomyrmex* have, in turn, lost their ability to digest sucrose^40^. Reciprocal adaptations such as these are likely common in mutualisms, as the number of interactions to which a mutualist must adapt is drastically increased.

Life histories must also be considered further. Although we are confident that the most frequent mechanisms causing higher rates of molecular evolution (i.e., generation time and population size) are not responsible for the differences in mutualistic plant-ants, several other processes are worth considering. The mechanism may be similar to that outlined above—that the interactions between ants and their hosts induce high rates of change—but rather than the continual changes throughout the evolutionary history of the relationship, these high rates may be due to the many changes required for the initial invasion into a new niche. For example, aggressive defense of their host plants is only one aspect of the convergent behavior of mutualists; they also have all evolved to feed predominantly on resources provided by their hosts, particularly the acacia-ants^40^,^41^. Alternatively, these host-derived diets may themselves lead to higher rates of molecular evolution by requiring energy intensive digestive processes that produce reactive molecules or may contain secondary metabolites that induce mutations at a higher rate than a less limited diet. Finally, the larger number of offspring produced by the mutualistic species, their longer lifespans, and the accompanying elevated number of germline DNA replications could lead to a higher rate of substitution^42^. Previous work has excluded this mechanism in social insects^43^ generally, though only a small amount of DNA sequence in relatively few advanced eusocial taxa was examined.

By sequencing and comparing seven ant genomes, we find that mutualistic plant-ants have higher rates of molecular evolution than closely related non-symbiotic species. This difference in rates has occurred in three different taxa that have convergently evolved mutualism and appears common among obligate symbionts^3^,^4^,^30^,^31^,^32^, suggesting that symbiosis is tied to evolutionary rate. Though we cannot, here, positively disentangle all potential mechanisms underlying the observed differences in rates of substitution between taxa, we expect that genomic analyses of additional, diverse mutualisms will show similar accelerations in evolutionary rates.

## Methods

### Genome sequencing

Ants were collected into 95% ethanol and stored at −20 °C. DNA was extracted using a standard phenol-chloroform procedure. Two Illumina libraries were used for *P. gracilis* genome assembly: a small insert ‘fragment' library and a mate-pair library. The fragment library was prepared from the DNA of a single haploid male specimen and was size-selected so that 100 base paired-end reads would overlap, as suggested by the authors of ALLPATHS-LG^17^. The mate-pair library was prepared from the remaining DNA available from the fragment library male combined with DNA from three of his brothers. The mate-pair library preparation was size-selected for 3 kb inserts. Each library was sequenced on its own Illumina HiSeq2000 lane.

The genome was assembled using ALLPATHS-LG release 44837 (ref. 17). Because our mate-pair library was prepared using DNA from multiple individuals, we ran the assembly pipeline twice. The first time, we set the PATCH_UNIPATHS, PATCH_SCAFFOLDS, and FIX_SOME_INDELS options to false to exclude the mate-pair reads from the part of the pipeline that uses them to patch holes in the assembly. We then ran the pipeline again, this time with default settings, but used the error corrected mate-pair reads resulting from the first run as input. These error-corrected reads were corrected to match the sequences from the fragment library, eliminating any heterozygosity introduced by multiple individuals. Therefore, the final genome assembly is equivalent to the haploid sequence from a single male.

We used CEGMA v2.4 (ref. 27) to assess the completeness of the assembled genome sequence. We used PILER-DF v1.0 (ref. 18) and RepeatModeler (http://www.repeatmasker.org) to predict the presence of TE sequences. CD-HIT (ref. 44) was used to reduce redundancy in the set of predicted TEs by representing sequences that were at least 80% similar over 80% of their length by the single longest sequence. We then aligned our TE predictions against the UniProt Swiss-Prot^45^ and *Drosophila melanogaster* proteins (FlyBase release FB2013_02) using BLASTX and removed predictions with bit scores of at least 100 or alignments of 50% similarity over 50% of sequence length as false positives^46^. We also removed TE predictions less than 80 bases long^46^. The remaining predictions were classified with the RepeatClassifier module of RepeatModeler. RepeatMasker (http://www.repeatmasker.org) and RepeatRunner^47^ were used by MAKER to generate a genome-wide repeat annotation based on the final set of TE predictions. The MAKER v2.30 (ref. 24) automated annotation pipeline was used to identify genes in the *P. gracilis* assembly. *Ab initio* gene predictions from both SNAP^48^ and AUGUSTUS^49^ were used in conjunction with the assembled ESTs and all annotated protein sequences from the ants *Acromyrmex echinatior* (OGS v. 3.8)^50^, *Atta. cephalotes* (OGS v. 1.2)^51^, *Camponotus floridanus* (OGS v. 3.3)^52^, *Harpegnathos saltator* (OGS v. 3.3)^52^, *Linepithema humile* (OGS v. 1.2)^53^, *Pogonomyrmex barbatus* (OGS v. 1.2)^54^, and *Solenopsis invicta* (OGS v. 2.2.3)^55^, honey bee, *Apis mellifera* (OGS v. 1.1)^56^, and jewel wasp, *Nasonia vitripennis* (OGS v. 1.2)^57^. All Hymenopteran gene sets were obtained from www.hymenopteragenome.org on April 3, 2013. SNAP and AUGUSTUS were initially trained on the 451 (of 458) CEGMA genes identified in the *P. gracilis* genome.

We ran standalone InterProScan version 5.2–45.0 (ref. 25) on all resulting gene models, including the predictions for which no transcript or protein evidence existed. If these predictions were associated with at least one gene ontology term, they were promoted to full models and included in the gene set. Sets of gene ontologies were also generated for *Acromyrmex echinatior*, *Atta cephalotes*, *Camponotus floridanus*, *Harpegnathos saltator*, *Linepithema humile*, *Pogonomyrmex barbatus*, *Solenopsis invicta*, *Apis mellifera*, and *Nasonia vitripennis.* We used the GO Term Finder software version 0.86 (ref. 58) to find gene ontology terms enriched within the *P. gracilis* annotation.

We used OrthoMCL version 2.0.9 (ref. 59) with default settings to find orthologues between the *P. gracilis* genes and the annotations from all Hymenoptera listed above as well as *Drosophila melanogaster*. When genes of interest had multiple possible orthologues or none found with OrthoMCL, we used BLASTP to find the most similar sequence in *Drosophila*.

### Transcriptome

RNA was extracted using the method of Alaux *et al*.^60^ with minor modifications. Briefly, ants were homogenized in 1 ml Trizol (Invitrogen Life Technologies, Grand Island, NY) and incubated at room temperature for 5 min. The solution was shaken vigorously for 15 s after adding 100 μl water and 200 μl chloroform, followed by another room temperature incubation for 3 min. The resulting mixture was then centrifuged for 15 min at 12,000 *g* at 4 °C. The aqueous phase was removed, mixed with an equal volume of 70% ethanol and transferred into a Qiagen RNeasy column for RNA extraction. DNA was eliminated with on-column DNase I (Qiagen, Valencia, CA) treatment.

Six RNA extractions were performed for *P. gracilis* transcriptome sequencing. Two of these included 10 workers, 2 included 10 larvae, 1 included 10 pupae and 1 included 3 pupae, ∼20 eggs and 6 larvae. All individuals used for RNA extractions were alive at the time of extraction. The RNA concentrations of these 6 extractions were determined with a NanoDrop2000 (Thermo Fisher Scientific, Waltham, MA) and were combined so that RNA from each extraction was equally represented in the final pool.

A standard library created from the RNA pool was sequenced on a single paired-end 100 base lane of the Illumina HiSeq2000 platform. Reads and their partners were eliminated from the data set if <90% of the read length had quality scores >20 using the FASTX-Toolkit (http://hannonlab.cshl.edu/fastx_toolkit/index.html). Remaining reads were assembled with the 25 February 2013 release of Trinity^61^ using default settings. Transcript redundancy was reduced using CD-HIT^44^ by clustering sequences at least 97% similar over 95% of their length.

### Annotation

Our initial annotations with the automated pipeline MAKER^24^ yielded a number of gene fusions; thus, we ran MAKER several times using different sets of evidence to reduce these anomalies. First, we executed MAKER with only protein sequences and *ab initio* predictors. The predictors were retrained on these results and re-run using the same data. We then executed MAKER again with the resulting models but with the addition of the transcriptome data to allow for the addition of untranslated regions to the protein-predicted genes. Lastly, we ran MAKER again with all available protein and transcript data. Our final gene set consisted of non-overlapping gene models from these separate MAKER runs with preference given to those models resulting from the protein-only-based models, except where two genes from the second set overlapped a single protein-only prediction. We also set the MAKER parameters ‘correct_est_fusion=1' and ‘always_complete=1', to further improve the annotation. Our approach successfully reduced the number of gene fusions present in the final gene set.

### NuMt identification

We queried the 13 protein sequence annotations from the complete *Solenopsis geminata* mitochondrial genome (HQ215537.1) against the assembled ant genomes using TBLASTN. The hit with the lowest *e*-value was used as the seed to identify nuclear mitochondrial-like copies of each gene in each genome. These seed nucleotide sequences were queried against the genome using BLASTN and hits with *e*-values <1 × 10^−20^ covering at least 35% of the length of the seed sequence were counted as introgressions of that particular gene.

### GBS sequencing

The queen and 47 workers of *P. gracilis* from a single colony in the same population as the individual whose genome was sequenced were genotyped using the GBS approach of Elshire *et al*.^19^ with an additional size selection step. As in Elshire *et al*.^19^, we used the restriction enzyme *Ape*KI and drew all adapter and barcode sequences, and the majority of the molecular techniques from that study. The barcoded adapters (5′-ACACTCTTTCCCTACACGACGCTCTTCCGATCTxxxx-3′ and 5′-CWGyyyyAGATCGGAAGAGCGTCGTGTAGGGAAAGAGTGT-3′, where ‘xxxx' and ‘yyyy' are the barcode sequences shown in Supplementary Data 2) and common adapters (5′-CWGAGATCGGAAGAGCGGTTCAGCAGGAATGCCGAG-3′ and 5′-CTCGGCATTCCTGCTGAACCGCTCTTCCGATCT-3′) were annealed at 50 μM by temperature ramping from 95 °C for 2 min down to 25 °C by 0.1 °C s^−1^ and holding at 25 °C for 30 min. Approximately 0.06 pmol of a barcoded and common adapter were combined with ∼200 ng of sample DNA and dried down overnight at 37 °C. Restriction digests were performed at 75 °C for 2 h in 20 μl volumes with 1 × NEB Buffer 3 and 3.6 U *Ape*KI (New England Biolabs, Ipswitch, MA). To these reactions, 30 μl of 1.66 × ligase buffer and 640 U T4 ligase (New England Biolabs) were added, followed by incubation at 22 °C for 1 h and heat inactivation at 65 °C for 30 min, to ligate adapters to restricted sample DNA. From these reactions, 20 μl of each sample was pooled and this pool was purified using a Qiagen QIAquick PCR purification kit. We size-selected for fragments 300–800 bp long using agarose gel electrophoresis and purified the sample with the Qiagen gel extraction kit, eluting in 30 μl. The restriction fragment library was then PCR amplified in 50 μl reactions with 2 μl of the pooled library, 2 μl of each primer (5′-AATGATACGGCGACCACCGAGATCTACACTCTTTCCCTACACGACGCTCTTCCGATCT-3′ and 5′-CAAGCAGAAGACGGCATACGAGATCGGTCTCGGCATTCCTGCTGAACCGCTCTTCCGATCT-3′) at 5 μM each, 21 μl of water and 25 μl of 2 × *Taq* master mix (New England Biolabs). Thermocycling consisted of 95 °C for 1 min followed by 18 cycles of 95 °C for 30 s, 65 °C for 30 s and 72 °C for 1 min with a final extension of 5 min at 72 °C. This reaction was replicated four times and the results pooled to reduce PCR biases. The pooled library was purified using a Qiagen QIAquick PCR purification kit and eluted in 30 μl. The result was sequenced directly on a single lane of an Illumina HiSeq2000 with 100 bp single-end reads.

### Linkage mapping

All GBS sequence processing was performed using the pyRAD^20^ pipeline. We expected restriction recognition site-associated sequences of CWGC for *Ape*KI. Default quality controls were used and reads were clustered at 90% similarity. We required that no more than three indels could be present in within sample clusters and at least 12 reads to be assigned to a within sample cluster.

We required that the queen was genotyped as heterozygous at each locus used in mapping analyses, as homozygous loci in the queen cannot contribute to the linkage analysis. Ants are haplodiploid; queens and workers are diploid and males are haploid. Typically, Hymenopteran linkage maps are produced by examining the haploid male offspring of a single queen. We instead examined the diploid worker offspring from a single nest. Therefore, each genotyped individual was either homozygous if the father had the same allele as the queen or heterozygous if those alleles differed. As we did not know the parental phase of the alleles at each locus, we followed the procedure of Wang *et al*.^21^. Briefly, we arbitrarily assigned the two alleles at each locus to different phases and added duplicates of these alleles with the phases reversed to the input data set. This resulted in a doubled number of linkage groups with two copies of each group consisting of the same loci with mirrored phases. One of the copies for each linkage group was arbitrarily discarded from the results.

We used MSTmap^22^ for linkage analysis with the Kosambi distance function and default error detection parameters (no_map_dist=15.0 and no_map_size=2). The missing data threshold was set to 25% and the cutoff *P*-value for locus linkage was set to 7.5 × 10^−6^ after some parameter exploration. We mapped loci that were successfully assigned to linkage groups to the genome sequence using the Burrows–Wheeler Aligner^62^ excluding alignments with gaps longer than three bases (-w 3) and discarding reads with more than one alignment in the genome (-c 1).

The resulting genotype calls were strictly filtered for inclusion in linkage map analysis. Samples were checked for excessive heterozygosity and those with large amounts of missing data were excluded. Loci were excluded if <75% of individuals were genotyped at that locus or if >2 alleles were present. The two alleles at each locus are expected to occur in approximately equal proportions. Deviations from this expectation may indicate the inclusion of paralogues in genotype calling or other types of misleading data. We therefore performed Fisher's exact tests on allele counts at all loci and those found to differ significantly from the expected 50% ratio were discarded.

### Sequencing of six ingroup *Pseudomyrmex* species

We sequenced a single diploid worker from mutualists *P. concolor*, *P. dendroicus* and *P. flavicornis*, and generalists *P. pallidus*, *P. elongatus* and the undescribed species *P.* sp. PSW-54. These species were chosen as representatives of mutualist/generalist sister clades within the genus based on Ward and Downie^13^, although the most probable phylogenetic positions of *P. concolor* and *P. pallidus* are different according to our results. We also sequenced an additional diploid worker of *P. gracilis* so that diploid genotypes were known for each species. Eight lanes of 100 bp paired-end Illumina HiSeq2000 sequencing were used, one for each species and two for *P. gracilis*. We estimated genome repetitiveness and single-nucleotide polymorphism rate of all diploid genomes from the raw data using k-mer spectrum analysis of the error correction module of ALLPATHS-LG^17^. Voucher specimens representing the species for which full genomes were sequenced have been deposited in the Field Museum's collection under accessions FMNHINS 2821891 to 2821900.

### Assembling *Pseudomyrmex* genomes

We used Stampy version 1.0.21 (ref. 63) to map reads from each additional *Pseudomyrmex* species to our *P. gracilis* assembly, specifying a 3% substitution rate between the reads and the mapping reference. We then used Platypus version 0.5.1 (ref. 64) to call genotypes of each species. We required there to be 5 × coverage to call genotypes, filtered PCR duplicate reads, allowed up to 30 variants in each window and merged variant containing windows. We also calculated coverage at all sites using the ‘mpileup' command of samtools^65^. We then used custom python scripts to merge these two sources of data into consensus sequences. Those sites for which Platypus did not yield genotype calls were called as the reference base only if they had 5 × or greater coverage. Otherwise, sites were called as unknown bases. When Platypus yielded >3 alleles at a site, they were also masked as unknown bases. We maintained the alignments of reads produced by Stampy and indel calls of Platypus, yielding a whole genome alignment of all seven *Pseudomyrmex* genomes. This alignment formed the basis of all genomic comparisons. Coordinates and reading frames of coding regions for all genomes were based entirely on the *P. gracilis* annotation.

### *Pseudomyrmex* phylogeny

We designed the taxonomic sampling of this research based on the phylogeny of Ward and Downie^13^ who showed that three pairs of mutualistic and generalistic lineages existed within *Pseudomyrmex*: *P. concolor* and *P. pallidus*, *P. dendroicus* and *P. elongatus*, and *P. flavicornis* and *P.* sp. PSW-54. However, before proceeding with our analyses, we aimed to confirm the phylogenetic relationships of each of these species. We therefore concatenated all scaffolds from each species and inferred a phylogeny using the threaded version of RAxML version 7.3.0 (ref. 66) and the GTRGAMMA model of evolution. We also inferred phylogenies from 25 kb sliding windows with step sizes of 5 kb, analysing only those windows for which all species had known sequence over at least half the window length.

### Tests for selection

We only examined signatures of selection in genes longer than 300 codons for which we obtained sequence from all seven species, requiring that each sequence had <20% missing data. Premature stop codons occurred in at least one sequence from ∼40% of genes that otherwise passed quality metrics. These stop codons typically were due to misalignments and were masked for all analyses. We used Gblocks version 0.91 (ref. 67) with default parameters to mask unconserved regions of gene nucleotide sequence alignments.

We used both the branch test and branch-site test implemented in PAML version 4.7 (ref. 37), to test for signatures of positive selection within the mutualistic lineages. We conservatively specified all three mutualists as ‘foreground' branches, reducing the chances of spuriously identifying genes under selection in just a single lineage and therefore not necessarily related to mutualism. We used the ‘cleandata' setting to remove ambiguous codons from sequences and used the topology recovered in this study as the species tree with the branch lengths from the total evidence whole-genome phylogeny as the initial estimate (fix_blength=1).

For the branch-site test, we first fit a model that allowed the mutualistic lineages to have a proportion of sites with dN/dS≥1, whereas the generalists had dN/dS≤1. This model was then compared with a second model that did not allow dN/dS to exceed one at any sites. The two models were compared using likelihood ratio tests and the resulting *P*-values were corrected to a 5% false discovery rate. We found that the branch-site test sometimes falsely identified genes under positive selection when they simply had high rates of substitution. To remedy this issue, we tested those genes that were found to be significant again, setting the non-mutualistic lineages as the foreground branches. Those that were detected to be under positive selection in both tests were discarded.

For the branch test, we again fit two models to the sequence data, setting the mutualistic lineages as the ‘foreground' branches and either allowing for the mutualists and non-mutualists to differ in estimated dN/dS ratios or fitting a model with just a single ratio across the tree. The resulting models were again compared with likelihood ratio tests and false discovery rate corrected. When possible, those genes found to be of interest were modelled structurally with Phyre2 (ref. 36) to determine the active sites of the proteins.

### Examining rates of molecular evolution

We assessed rates of molecular evolution of each sequenced species using a number of approaches, all of which were based on pairs of most closely related mutualists and generalists. It is noteworthy that we considered *P. concolor* and *P. pallidus* to be a pair of mutualist and generalist for all analyses, although, given our phylogenetic findings, other taxa were equally closely related. Most basically, we calculated genetic distance of each species from *P. gracilis* using the same sliding windows used to create phylogenies from across the genome. We assessed the statistical significance of differences in genetic distances using paired *t*-tests, pairing distances from the same windows. We also counted the number of windows for which mutualists had consistently greater genetic distances from *P. gracilis* than the most closely related generalists. We used a similar approach with the branch lengths recovered from the inferred phylogenies of each window.

We also estimated rates of non-synonymous and synonymous substitutions in all genes using the free-ratios model implemented in PAML. Again, we used the species tree from this study and used ‘cleandata=1' to mask ambiguous data. Only those genes with dN/dS ratios <10 were examined as larger values were very likely to be due to assembly or annotation errors. Wilcoxon signed-rank tests were used to compare overall rates of change between species. We confirmed the quality of our overall estimates of dN/dS by summing all dN and dS estimates within each species and calculating the ratio based on these sums, thus reducing the influence of outliers.

### Classifying and quantifying repetitive elements

We randomly subsampled 100,000 paired-end sequences from the raw data for each species using seqtk (https://github.com/lh3/seqtk). We then ran Transposome^68^ on these data with a merge threshold of 0.0001. All other parameters were defaults. Clustered sequences were classified by Transposome using the repeat database built from the *P. gracilis* genome.

### Codon usage

Codon usage bias can be a result of selection, in particular when genes are highly expressed and translation efficiency is influential to their production. We measured codon usage bias by estimating effective codon number with ENCprime^69^ using the 2,000 bases flanking each end of a given gene to estimate background nucleotide composition. In addition to examining correlations between effective number of codons and the rates of non-synonymous and synonymous changes in each genome estimated by PAML, we also estimated the same parameters using HyPhy^38^. We tested for differences in genome-wide codon bias between taxa using Wilcoxon rank-sum tests.

### Duplications

To determine whether particular genes have been duplicated in mutualistic lineages, we mapped reads from each species to the *P. gracilis* genome using mrFAST version 2.6.0 (ref. 70) allowing 6% edit distance. The genome was first repeat-masked using RepeatMasker version 4.0.1 (http://www.repeatmasker.org). We examined the resulting mappings with mrCaNaVaR version 0.51 (ref. 70). We then looked for regions where estimated sequence copy number was at least 1.5 and all of the species with each life history were consistently at least 1.5 × higher than all species with the other life history. In particular, we focused on coding regions that showed these patterns.

### Signatures of parallel evolution

We used Saguaro version 0.1 (ref. 28) with default settings to search for signatures of parallel evolution within the mutualistic lineages, first filtering for those sites where non-gap calls had been made in at least two mutualists and two generalists. We also examined the topologies resulting from our sliding window analyses of phylogenetic relationships to determine whether any parts of the mutualistic genomes appeared more closely related to each other than to any generalists.

### Estimating population sizes

We estimated *θ* for each of the ingroup species using G-PhoCS^33^. Genomic regions found to be repetitive by RepeatMasker (http://www.repeatmasker.org) in the *P. gracilis* reference genome were masked from the alignment, as were all coding sequences and the flanking 5,000 bases, to increase the odds of obtaining neutrally evolving sequences. We then sampled all loci of length 500 at least 15 kb apart with sequence data for at least four of the six in-group species. Sequence was not considered present in a given species if >20% of the total length consisted of undetermined sequence or gaps.

The first 1,000,000 iterations of the G-PhoCS MCMC run were discarded as burn-in and values were sampled for 2,000,000 iterations every 100 iterations thereafter for a distribution of 20,001 samples. These distributions were manually inspected for convergence.

### Expression levels of fast-evolving genes

We determined the set of genes with consistently higher rates of molecular evolution in mutualistic lineages using the phylogenetic framework outlined above. Those genes for which all three mutualistic lineages in each pair of species (*P. concolor* versus *P. pallidus*, *P. flavicornis* versus *P.* sp. PSW-54 and *P. dendroicus* versus *P. elongatus*) had greater rates of molecular evolution were considered to be fast-evolving in mutualists. Those genes that showed the opposite pattern, with rates of change consistently higher in generalists, were used for comparison, as were the genes with no consistent pattern between mutualists and generalists. We used modENCODE's library of *Drosophila* tissue gene expression data to determine whether these fast-evolving genes are expressed at different levels in particular tissues. There are 29 tissue types included in the modENCODE data set. For all genes for which a *Drosophila* orthologue was found using OrthoMCL, modENCODE expression levels were standardized to one across all tissues. Within each tissue, the level of expression of the fast-evolving genes was compared with standardized expression of all other genes using *t*-tests.

### Selection on aggression genes

We expected genes known to be involved in aggression in other social Hymenoptera to be over-represented in the genes we identified as being under convergent selection or evolving at higher rates in mutualists due to their highly aggressive behaviour. Therefore, we assembled lists of genes putatively involved in aggression in honey bees^39^. The first list consists of 51 genes confidently determined to be involved in aggression. The second list includes 1,922 genes, all of which were implicated in aggression in some way. We identified orthologues in *Pseudomyrmex* and looked for over-representation of fast-evolving genes among these aggressiveness genes.

### Data availability

All of the raw sequence data that support the findings of this study have been deposited in the National Center for Biotechnology Information's Whole Genome Shotgun database under BioProject PRJNA268384 along with the *P. gracilis* assembly. *Pseudomyrmex gracilis* annotations and aligned reference-based assemblies for all species are available from the Dryad Digital Repository at http://dx.doi.org/10.5061/dryad.q530s as well as at http://benrubin.science/data/. All other data necessary to obtain our results were obtained from http://hymenopteragenome.org and http://flybase.org.

## Additional information

**How to cite this article:** Rubin, B.E.R. & Moreau, C.S. Comparative genomics reveals convergent rates of evolution in ant-plant mutualisms. *Nat. Commun.* 7:12679 doi: 10.1038/ncomms12679 (2016).

## Supplementary Material

Supplementary Information Supplementary Tables 1 - 7

Supplementary Data 1 Repetitive elements in the *P. gracilis* genome.

Supplementary Data 2 Genotyping-By-Sequencing results.

Supplementary Data 3 Details of linkage map.

Supplementary Data 4 Gene ontology terms enriched in unique *P. gracilis* genes.

Supplementary Data 5 Gene ontology terms enriched in the *P. gracilis* genome compared to other Hymenoptera.

Supplementary Data 6 Counts of all topologies recovered from 25kb sliding windows.

Supplementary Data 7 Duplicated genic regions with consistently larger numbers of copies in mutualists or generalists.

Supplementary Data 8 Genes with signatures of positive selection in mutualists.

Supplementary Data 9 T-tests comparing gene expression levels of genes with consistently greater dS in mutualists and generalists in a variety of *Drosophila* tissues.

Supplementary Data 10 T-tests comparing gene expression levels of genes with consistently greater dN ratios in mutualists and generalists in a variety of *Drosophila* tissues.

Supplementary Data 11 T-tests comparing gene expression levels of genes with consistently greater dN/dS ratios in mutualists and generalists in a variety of *Drosophila* tissues.

Supplementary Data 12 T-tests comparing gene expression levels of genes with consistently greater genetic distance in mutualists and generalists in a variety of *Drosophila* tissues.

Supplementary Data 13 Proportion of all codons used by each species.
