# Supplementary material for: Comparative genomics reveals convergent rates of evolution in ant–plant mutualisms
Source: Nat Commun. 2016 Aug 25;7:12679. doi: 10.1038/ncomms12679 (PMC5007375; doi:10.1038/ncomms12679)
Supplement: Supplementary Information — Supplementary Tables 1 - 7 [file ncomms12679-s1.pdf]

Supplementary Tables

Supplementary Table 1. Counts of mitochondrial-like sequences identified in *P. gracilis* and seven other ant genomes.

Mitochondrial gene	*P. gracilis*	*A. cephalotes*	*A. echinatior*	*P. barbatus*	*S. invicta*	*C. floridanus*	*L. humile*	*H. saltator*
ATP6	**33**	32	4	4	2	2	3	1
ATP8					1			
COI	**54**	14	5	3	2	2	3	1
COII	7	**13**	5	8	1	2	1	1
COIII	9	**19**	3	12	4	2	4	1
Cytb	**61**	45	4	6	2	1	3	2
ND1	10	2	3	**11**	3	1	2	1
ND2	5	1	3	**8**	2	2		
ND3	**21**	13	3	10	1	1	2	1
ND4	**8**	3	5	**8**	4	2	2	1
ND4L	4	**21**	1	17	1	1	2	1
ND5	**16**	10	6	6	3	3	6	1
ND6				4	2			
Total	228	173	42	97	28	19	28	11

Greatest counts of each gene are in bold.

Supplementary Table 2. Reference-based assembly statistics.

Species	Repetitiveness	SNP rate	#Individual reads	Mapped reads	Genome bases called	#Genes with >80% sequence	GC content	Mean GC3	#TE families	Max genome fraction from individual TE family
P. gracilis	41%	1/276	548,112,030	538,986,494	92.60%	16,004	39.1%	50.5%	1220	0.007917
P. concolor	27%	1/755	359,475,424	314,250,463	66.30%	13,234	39.0%	49.4%	693	0.03565
P. pallidus	39%	1/612	342,184,040	284,653,013	66.70%	13,491	39.6%	50.4%	708	0.01374
P. flavicornis	32%	1/576	290,503,558	248,888,387	65.80%	13,213	39.2%	50.0%	660	0.01641
P. sp. PSW-54	32%	1/930	368,563,850	316,350,757	66.50%	13,258	39.4%	50.4%	690	0.01393
P. dendroicus	31%	1/550	366,341,280	317,075,125	67.20%	13,399	39.2%	49.6%	723	0.02982
P. elongatus	36%	1/1,185	409,687,406	331,499,186	67.90%	13,593	39.4%	50.2%	756	0.01501

Repetitiveness and SNP rate were estimated by ALLPATHS-LG from raw data.

Supplementary Table 3. Paired t-tests and Wilcoxon signed-rank tests of rates of molecular evolution between all pairs of mutualists and generalists.

		25 kb genomic windows		CDS		Branch lengths		dN		dS		dN/dS		Nc	
Mutualist	Generalist	*T* value	*P* value	*T* value	*P* value	*T* value	*P* value	*U* value	*P* value	*U* value	*P* value	*U* value	*P* value	*U* value	*P* value
P. concolor	*P. pallidus*	53.4	0.00	5.0	7.60E-07	25.7	7.59E-144	8.7E06	0.780	9.1E06	5.35E-7	-8.3E06	0.0333	3.4E06	0.00869
P. flavicornis	*P.* sp. PSW-54	47.1	0.00	6.8	9.95E-12	353.7	0.00	9.1E06	1.64E-8	1.1E07	7.07E-89	-8.1E06	2.14E-5	3.4E06	0.175
P. dendroicus	*P. elongatus*	67.2	0.00	4.5	6.12E-06	180.6	0.00	8.8E06	0.0151	9.3E06	2.14E-11	-8.5E06	0.433	3.3E06	0.449
P. concolor	*P.* sp. PSW-54	69.2	0.00	14.0	3.62E-44	62.0	0.00	9.6E06	1.67E-24	1.2E07	2.12E-164	-8.2E06	6.69E-4	3.5E06	3.63E-06
P. concolor	*P. elongatus*	100.1	0.00	11.5	1.28E-30	51.1	0.00	9.3E06	1.63E-11	9.9E06	2.35E-37	8.6E06	0.559	3.4E06	0.0268
P. flavicornis	*P. pallidus*	20.7	1.21E-92	-2.6	1.00E-02	139.5	0.00	-8.1E06	3.81E-5	-8.2E06	7.69E-3	-8.2E06	3.50E-3	3.3E06	0.5305
P. flavicornis	*P. elongatus*	67.8	0.00	4.0	5.48E-05	158.9	0.00	8.8E06	0.0165	9.1E06	6.18E-7	-8.5E06	0.836	3.2E06	0.2886
P. dendroicus	*P. pallidus*	5.9	4.54E-09	-2.1	3.50E-02	65.2	0.00	-8.1E06	4.88E-5	-8.4E06	0.303	-8.2E06	4.64E-4	3.3E06	0.2301
P. dendroicus	*P.* sp. PSW-54	22.7	1.17E-110	7.2	4.66E-13	138.0	0.00	9.1E06	1.88E-8	1.1E07	4.77E-104	-8.0E06	1.27E-6	3.3E06	0.2301

Nc is the effective number of codons as calculated by ENCprime

Supplementary Table 4. Counts of genomic features with greater rates of evolution in mutualists and generalists.

		25 kb genomic windows		CDS		Branch lengths		dN		dS		dN/dS	
Mutualist	Generalist	Mutualist greater	Generalist greater	Mutualist greater	Generalist greater	Mutualist greater	Generalist greater	Mutualist greater	Generalist greater	Mutualist greater	Generalist greater	Mutualist greater	Generalist greater
P. concolor	*P. pallidus*	5892	2060	6159	4478	20061	18801	2055	2038	2243	1885	1952	2179
P. flavicornis	*P.* sp. PSW-54	5750	2202	6943	3295	37293	1569	2480	1379	3376	746	1671	2459
P. dendroicus	*P. elongatus*	6279	1673	6142	4233	32415	6447	2245	1787	2453	1676	2033	2098
All pairs		3580	206	2364	620	16801	235	775	347	1174	173	443	707
All three		1583	44	1106	261	8495	45	246	79	304	26	114	171

25 kb windows and CDS are counts of genetic distances from *P. gracilis.*

Supplementary Table 5. Estimates of θ from G-PhoCS (x1E-4)

	Mean	SD	95% HPD
P. concolor	29.83	0.49	28.89, 30.83
P. pallidus	30.38	0.48	29.48, 31.35
P. flavicornis	23.31	0.39	22.56, 24.08
P. sp. PSW-54	26.58	0.42	25.77, 27.43
P. dendroicus	30.42	0.49	29.49, 31.38
P. elongatus	25.54	0.42	24.74, 26.39

Supplementary Table 6. Genes with at least two convergent amino acid sites in all mutualists or all generalists.

Convergence	Gene	*Drosophila* ortholog	Gene name
mutualists	PG01652	FBgn0036892	Lon protease
mutualists	PG07059	FBgn0264695	Myosin heavy chain
mutualists	PG07966	FBgn0265045	Stretchin-Mlck
mutualists	PG12168		
mutualists	PG11122	FBgn0033342	CG8258
mutualists	PG03547	FBgn0052206	CG32206
mutualists	PG11682		
mutualists	PG13219	FBgn0051028	CG31028
mutualists	PG07438	FBgn0040001	CG17374
mutualists	PG05568		
mutualists	PG07577	FBgn0035842	CG7504
mutualists	PG09838		
mutualists	PG09393	FBgn0033451	CG1665
generalists	PG06208	FBgn0014018	Relish
generalists	PG10504		
generalists	PG04631	FBgn0032026	CG7627
generalists	PG13448		
generalists	PG02367		
generalists	PG12450	FBgn0262517	l(3)76BDr
generalists	PG01465		
generalists	PG05101	FBgn0085398	pickpocket 9
generalists	PG08252	FBgn0037882	CG17187

Supplementary Table 7. Correlations between rates of molecular evolution and effective number of codons.

	PAML estimates of rates of molecular evolution								
	dN			dS			dN/dS		
Species	*T* value	*P* value	*r*	*T* value	*P* value	*r*	*T* value	*P* value	*r*
P. concolor	5.69	1.37E-08	9.41E-02	-11.35	2.20E-16	-1.77E-01	6.61	4.48E-11	1.09E-01
P. pallidus	3.89	1.01E-04	6.64E-02	-5.86	5.05E-09	-9.40E-02	4.77	1.95E-06	8.14E-02
P. flavicornis	5.09	3.91E-07	9.71E-02	-8.83	2.20E-16	-1.44E-01	3.81	1.41E-04	7.05E-02
P. sp. PSW-54	2.26	2.37E-02	4.69E-02	-6.42	1.56E-10	-1.11E-01	2.15	3.15E-02	4.27E-02
P. dendroicus	5.20	2.10E-07	9.25E-02	-7.05	2.08E-12	-1.14E-01	3.51	4.59E-04	6.21E-02
P. elongatus	4.63	3.82E-06	8.63E-02	-4.31	1.71E-05	-7.09E-02	1.89	5.83E-02	3.50E-02
	HyPhy estimates of rates of molecular evolution								
	dN			dS			dN/dS		
	T value	*P* value	*r*	*T* value	*P* value	*r*	*T* value	*P* value	*r*
P. concolor	2.80	5.19E-03	6.02E-02	0.61	5.41E-01	1.09E-02	3.12	1.82E-03	7.34E-02
P. pallidus	0.59	5.55E-01	1.23E-01	-2.02	4.31E-02	-3.47E-02	2.76	5.81E-03	6.14E-02
P. flavicornis	0.54	5.92E-01	1.19E-02	-2.22	2.63E-02	-4.00E-01	1.22	2.22E-01	3.05E-02
P. sp. PSW-54	2.07	3.83E-02	4.15E-02	-2.11	3.53E-02	-3.48E-02	3.38	7.49E-04	7.13E-02
P. dendroicus	2.47	1.35E-02	5.07E-02	0.37	7.14E-01	6.14E-03	1.85	6.39E-02	4.06E-02
P. elongatus	2.85	4.42E-03	5.26E-02	-3.54	4.00E-04	-5.78E-02	3.18	1.47E-03	6.07E-02
